# The complete chloroplast genome sequence of *Trollius macropetalus*

**DOI:** 10.1080/23802359.2021.2005475

**Published:** 2021-11-29

**Authors:** Dejiang Liu, Jian Shen, Wenjing Fan, Chengjun Yang, Jun Xia, Lili Li

**Affiliations:** a China-Ukraine Joint International Research laboratory for Agriculture&Forestry Technology Development and Application, Jiamusi University, Jiamusi, China; b School of Forestry, Northeast Forestry University, Harbin, China

**Keywords:** *Trollius macropetalus*, chloroplast genome, phylogenetic analysis

## Abstract

The complete chloroplast genome of *Trollius macropetalus* was sequenced in this study. It has a cyclic tetrad structure typical of angiosperms. The total length is 160,094 bp, including a large single copy region (LSC) with a length of 88,555 bp, a small single copy region (SSC) with a length of 18,291 bp and two equal-length inverted repeat regions (IRA/IRB) with a length of 26,624 bp. It encodes a total of 137 genes, including 87 protein-coding genes, 42 tRNA genes, and 8 rRNA genes, with a CG content of 38.03%.

*Trollius macropetalus* (Regel) F. Schmidt is belongs to the Subfam. Helleboroideae (Lei et al. 2019). It is widely distributed and prefers cold and humid environment. It is mainly distributed in Shanxi, Hebei, Liaoning, Heilongjiang province and other northern parts of China (Zhu et al.^2006^). It is a garden ornamental plant. And it has medicinal functions such as heat-clearing and detoxification, anti-inflammatory and sterilization. It is also edible, and its taste is spicy (Li 2017). This study completed the sequencing of the chloroplast genome of *Trollius macropetalus* and enriched the genetic information of *Trollius macropetalus*. In the phylogenetic analysis of the Subfam. Helleboroideae, the position of *Trollius macropetalus* in the systematic genetic evolution was determined. It laid a theoretical foundation for the research of molecular breeding, evolution analysis and phylogeny of *Trollius macropetalus* in the later period.

The experimental materials were collected from the agricultural and forestry experiment and demonstration base of Jiamusi university in China (46°74′–46°75′N, 130°35′– 130°36′E). The voucher specimen (JMS20200609TM01) was deposited at Herbarium, Jiamusi University, Jiamusi city, China. After collecting the fresh leaves, immediately wrap them in tin foil and store them in liquid nitrogen for later use. The whole genomic DNA of *Trollius macropetalus* leaves was extracted with a plant genomic DNA extraction kit (Dong 2018). Sequencing with Illumina HiSeq2500 to generate the original sequence. To ensure the quality of subsequent information analysis, the original data is further filtered and saved in FASTQ format (Zhang et al. 2016). The genome of *Trollius macropetalus* was assembled using BioEdit software. CPGAVAS software (Liu et al. 2012) was used to annotate genes in the chloroplast genome of *Trollius macropetalus*. In this study, the complete chloroplast genome sequence of *Trollius macropetalus* has been deposited in the Gene Bank under the accession number MW308598.

The complete chloroplast genome of *Trollius macropetalus* was 160,094 bp in length, including a large single copy region (LSC) with a length of 88,555 bp, a small single copy region (SSC) with a length of 1,8291 bp and two equal-length inverted repeat regions (IRA/IRB) with a length of 26,624 bp. It encoded a total of 137 genes, including 87 protein-coding genes, 42 tRNA genes, and 8 rRNA genes, with a CG content of 38.03%. Phylogenetic analysis uses the complete chloroplast genomes of 11 species of Subfam. Helleboroideae reported in GenBank of NCBI and takes *Salix wilsonii* as an outgroup. All of the cp genome sequences were aligned using the program MAFFT version 7 (Katoh et al. 2005) and adjusted manually where necessary. Maximum likelihood (ML) analyses were implemented in RAxML version 8.2.12 (Stamatakis 2014). RAxML searches relied on the general time reversible (GTR) model of nucleotide substitution with the gamma model of rate heterogeneity. Non-parametric bootstrapping as implemented in the fast bootstrap algorithm of RAxML used 1000 replicates.

The results showed that *Trollius* is in a small branch. It can be seen from the figure that the *Trollius chinensis* has the closest relationship with the *Trollius macropetalus* and the *Trollius farreri* has the closest relationship with the *Trollius ranunculoides* (Figure 1).

**Figure 1.**
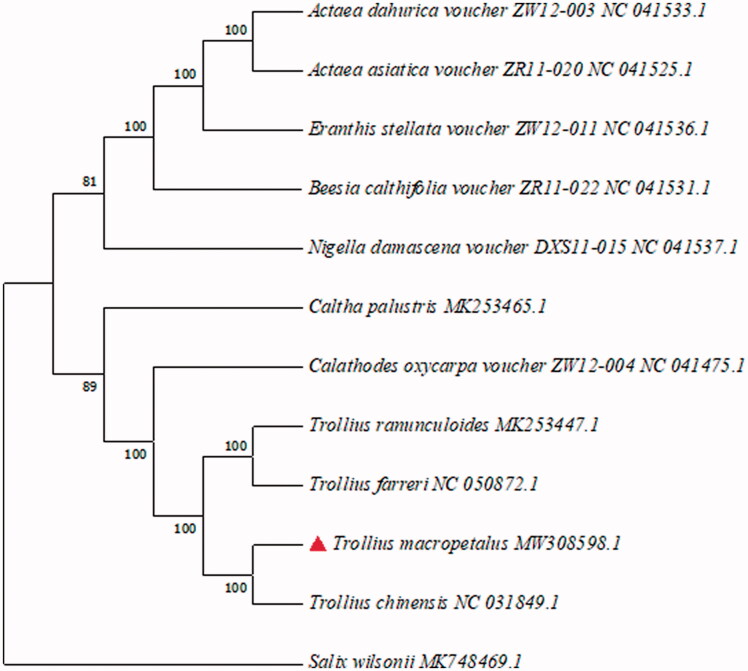
Phylogenetic tree inferred by maximum likelihood (ML) method based on the complete chloroplast genome of 11 species of Subfam. Helleboroideae and taking *Salix wilsonii* as an outgroup.

## Data Availability

The genome sequence data that support the findings of this study are openly available in GenBank of NCBI at https://www.ncbi.nlm.nih.gov/nuccore/MW308598. The SRA numbers is SRX12678191 at https://www.ncbi.nlm.nih.gov/sra/SRX12678191[accn].

## References

[CIT0001] Dong YT. 2018. Study on Ginkgo biloba chloroplast genome. （Master Dissertation）Nanjing Forestry University. pp. 24–28.

[CIT0002] Katoh K, Kuma K-I, Toh H, Miyata T. 2005. MAFFT version 5: Improvement in accuracy of multiple sequence alignment. Nucleic Acids Res. 33(2):511–518. 15661851 10.1093/nar/gki198 PMC548345

[CIT0003] Lei H, Zhu SJ, Zhao Y, Duan XX. 2019. Sequencing and characteristic analysis of chloroplast genome of *Trollius chinensis*. Genom Appl Biol. 8:3595–3604.

[CIT0004] Li XL. 2017. Development and utilization value and cultivation techniques of *Trollius chinensis*. China Agric Abst Agric Eng. 29(04):73–74.

[CIT0005] Liu C, Shi L, Zhu Y, Chen H, Zhang J, Lin X, Guan X. 2012. CPGAVAS, an integrated web server for the annotation, visualization, analysis, and GenBank submission of completely sequenced chloroplast genome sequences. Bmc Genomics. 13(01):715. 23256920 10.1186/1471-2164-13-715 PMC3543216

[CIT0006] Stamatakis A. 2014. RAxML version 8: a tool for phylogenetic analysis and post-analysis of large phylogenies. Bioinformatics. 30(9):1312–1313. 24451623 10.1093/bioinformatics/btu033 PMC3998144

[CIT0007] Zhang YW, Li W, Zhang LF, Wang CJ, Dai HY, Xu R. 2016. Whole genome variation mining of new soybean variety Qi Huang 34 based on resequencing. Chinese J Oil Crops. 38(2):150–158.

[CIT0008] Zhu DL, Ding WL, Chen SL. 2006. Research progress of *Trollius*. World Sci Technol. 8(4):26–33.

